# Effects of intermittent pneumatic compression on the recovery of cardiovascular parameters after repeated sprint exercise

**DOI:** 10.1007/s00421-023-05333-x

**Published:** 2023-10-04

**Authors:** Arnau Artés, Pau Ferrer-Ramos, Casimiro Javierre, Ginés Viscor, Iker García

**Affiliations:** 1 Departament de Biologia Cellular, Fisiologia I Immunologia, Facultat de Biologia, Universitat de Barcelona, Av. Diagonal, 643, 08028 Barcelona, Spain; 2 Department of Health Sciences, Research group in Technology Applied to high performance and health, Universitat Pompeu Fabra, Av. d’Ernest Lluch, 32, 08302 Mataró, Spain; 3 Departament de Ciències Fisiològiques, Facultat de Medicina, Universitat de Barcelona, Feixa Llarga s/n, 08907 Hospitalet de Llobregat, Spain

**Keywords:** Pneumatic compression, Cardiovascular system, Sports recovery, Sprint interval training, Blood pressure, Heart rate

## Abstract

**Purpose:**

Intermittent pneumatic compression (IPC) applies gradual pressure to facilitate lymph and blood flow movement to reduce exercise-induced tissue fluid accumulation and plasma volume loss. This study aimed to evaluate the cardiovascular system response during the recovery with IPC compared with passive recovery (Sham).

**Methods:**

Sixteen volunteers (7 females and 9 males) executed a cycling-based exhausting sprint interval exercise (8 × 20 s all out), followed by a 30-min IPC or Sham condition. Participants performed two trials in a randomised, counterbalanced, and crossover design. Several cardiovascular parameters (blood pressure, heart function, and peripheral vascular resistance) were recorded at baseline (5ʹ), through the recovery protocol (30ʹ), and afterwards (5ʹ).

**Results:**

The use of IPC during the recovery phase led to a faster recovery, stated in relative values to pre-exercise, in mean blood pressure (102.5 ± 19.3% vs. 92.7 ± 12.5%; P < 0.001), and cardiac output (139.8 ± 30.0% vs. 146.2 ± 40.2%; P < 0.05) in comparison to Sham condition. Furthermore, during the IPC-based recovery, there was a slower recovery in cardiac pressure change over time (92.5 ± 25.8% vs. 100.5 ± 48.9%; P < 0.05), and a faster return to pre-exercise values in the peripheral vascular resistance (75.2 ± 25.5% vs. 64.8 ± 17.4%; P < 0.001) compared to Sham.

**Conclusion:**

The application of IPC after high-intensity exercise promotes the recovery of the cardiovascular system, reducing cardiovascular strain. Future investigations should consider the effects on the sympathetic-parasympathetic balance, such as heart rate variability, to assess further bonds between the use of IPC and autonomous control.

## Introduction

Intermittent pneumatic compression (IPC) is an extensively used therapeutic strategy in vascular medicine, which consists of gradual pressure gradients applied to facilitate lymph and blood flow (Morris et al. 2008). Venous blood return depends on lower limb muscle tone, body position, and bicuspid valve functionality to prevent blood reflux (Williams et al. 2014).

The first modern pneumatic compression system described in the literature was the “CollWil pump”, which provided a simple uniform thigh compression at about 80 mmHg, in cycles of 2-min or 4-min compression appearing to improve symptoms in a range of arterial diseases (Morris 2008). IPC is thought to be a mechanical “squeezing” of the limb that facilitates swelling out of the extremity and increase blood flow, mimicking muscle contraction-induced increase of venous return (Morris 2008).

Regarding haemodynamic and cardiovascular changes, the application of IPC has been shown to produce hyperaemia during the decompression of the extremity treated, followed by a reduction of the blood flow during the compression phase (Morris and Woodcock 2002; Roseguini et al. 2010). This physiological dynamic could be associated with an improvement of compressed-zone resistance vessel reactivity and the endothelial function of systemic conduit arteries (Martin et al. 2015a), as well as an increase in the expression of angiogenic factors, such as vascular endothelial growth factor, and monocyte chemoattractant protein-1 (Roseguini et al. 2010).

Sports practitioners have included IPC as a recovery method, raising its popularity, due to the observed benefits, including the reduction of fluid accumulation in the tissue, as well as the increase in venous and lymphatic return (Morris 2008; Cochrane et al. 2013; Williams et al. 2014). The application of moderate compression (i.e. 50 mmHg) has been associated with increased cardiac output and systolic volume, and a reduction of heart rate and peripheral vascular resistance (Bickel et al. 2011). Moreover, some studies have observed favourable effects of IPC on flexibility (Sands et al. 2014), muscle soreness (Sands et al. 2015), muscle swelling and stiffness (Chleboun et al. 1995) and lactate removal rate compared to passive recovery, but without differences with active recovery (Hanson et al. 2013; Martin et al. 2015b). Despite this, it does not seem to have a positive effect on subsequent athletic performance (Overmayer and Driller 2018) and post-exercise recovery biomarkers of muscle damage such as blood creatine kinase levels (Cochrane et al. 2013).

As far as we are aware, the effects of IPC on cardiovascular dynamics have been studied following submaximal exercise or at rest (Bickel et al. 2011; Khan et al. 2021). Thus, this work aims to evaluate the effects on cardiovascular physiology of acute IPC application during the recovery period following an all-out intensity exercise. Several cardiovascular parameters will be evaluated pre-exercise, during the recovery phase, and immediately after the recovery. We hypothesized that using IPC would accelerate the recovery of the studied heart and circulatory parameters during the recovery phase compared to the Sham condition.

## Materials and methods

### Participants

Sixteen subjects were recruited, including 7 females and 9 males (mean ± standard deviation; 27.7 ± 9.4 years; 175.8 ± 9.8 cm; 69.0 ± 12.0 kg; BMI 22.3 ± 2.9 kg·m^−2^). All subjects were healthy and trained, regularly engaging in high-intensity interval exercise with at least 3 sessions per week. Being habituated to high-intensity exercise and not presenting medical contraindications were also criteria for inclusion. The study was developed in accordance with the Declaration of Helsinki concerning the ethical principles of human experimentation and approved by the Institutional Ethical Committee from the University of Barcelona (Institutional Review Board no. IRB00003099). All participants provided informed written consent before taking part in the study and were free to withdraw from the experimental protocol at any time.

### Experimental design

This study used a randomized, counterbalanced, crossover design to observe the effects of IPC in post-exercise recovery compared with passive recovery (Sham) condition. Participants reported to the laboratory on two separate occasions, separated by 7 days, to perform two trials (IPC or Sham) in a randomised, counterbalanced, crossover design to evaluate the effect of IPC in post-exercise recovery. Participants were instructed to arrive in a rested, hydrated, postprandial state (> 2 h) and to avoid caffeine, alcohol, and strenuous exercise in the 24 h preceding a session. Participants were also instructed to maintain normal dietary habits throughout the study and replicate their 24-h diet for subsequent visits.

Upon arrival at the laboratory, they were asked to empty their bladders. All testing was performed on the same cycle ergometer (Concept2, Morrisville, Vermont, United States), and at the same time of day (± 1 h), to minimise chronobiological variations. During the recovery protocol, participants were randomly assigned to either IPC or Sham on the first visit and to the other condition in the following week.

### Procedures

Baseline measurements of cardiovascular variables (blood pressure, heart function, and peripheral vascular resistance) were collected in a supine position during 5 min of resting period (pre-exercise). Then, a repeated sprint exercise (RSE) was performed. This exercise modality is based on short (≤ 30 s), all-out intensity efforts interspersed with resting or light to moderate-intensity recovery periods lasting from 1 to 4 min (Buchheit and Laursen 2013; Jiménez-Maldonado et al. 2020). Immediately after the exercise, subjects lied down on a stretcher in the supine position to perform the recovery protocol (IPC or Sham) in a randomised crossover design, starting 5 min after the end of the exercise. The recovery protocol was applied in a resting, supine position for 30 min. The Sham recovery condition consisted of using the IPC “leg sleeves” connected to the pneumatic pump but was devoid of actual pressure. This protocol is employed to control for any thermogenic effect of wearing the legs sleeves as heat loss from the legs is likely relevant during a 30-min recovery phase. Starting 2 min after the end of the recovery, the leg sleeves were removed, and 5 min of a resting period in the same supine position were recorded (post-recovery).

### Exercise protocol

Following baseline measurements, the cycle ergometer seat height and position were adjusted for the subject, before beginning a 10-min warm-up period by pedalling against self-selected air resistance and cadence of 70 rpm producing 1 W/kg for 5 min, 1.5 W/kg for 3 min and 2 W/kg for 2 min. One minute afterwards, they started the RSE, consisting of eight 20 s all-out efforts pedalling against maximal air resistance interspersed with 1 min of passive recovery (i.e., without pedalling). Participants were asked to perform the eight repetitions at the maximal intensity possible from the first one. The exercise protocol was executed in a separate cycle ergometer equipped with an electronic computer displaying the effort and recovery time but blinding the watts and the cadence produced by the subject during the exercise.

### Intermittent pneumatic compression

An intermittent sequential pneumatic compression device (Recovery Air 3 PRO, Therabody®, Los Angeles, CA) was used for IPC treatments. They are commonly used on an individual basis by endurance athletes, as well as in a clinical setting by athletic trainers and clinicians. The pneumatic compression device consists of 2 separate “leg sleeves”, which contain 4 circumferential inflatable chambers (arranged linearly along the limb) encompassing the leg from the feet to the hip/groin. The “leg sleeves” are connected to an automated pneumatic pump at which target inflation pressures for each zone and the duty cycle can be controlled. The compression modality was sequential, where a single pressure is applied to parts of the limb in a distal-to-proximal sequence with 4 overlapped chambers per sleeve, creating a negative pressure gradient from distal to proximal. In this study, the compression applied to the most distal chamber was 80 mmHg, declining 1 mmHg in the subsequent proximal cells, establishing a negative pressure of 80, 79, 78, and 77 mmHg in each chamber respectively.

### Finger photoplethysmography

Finger photoplethysmography (Nexfin™; BMEYE B.V., Amsterdam, Netherlands) was used for the continuous measurement of all cardiovascular variables, based on the volume-clamp method as proposed by Peňáz (Martina et al. 2012). Briefly, the Nexfin device is based on a finger cuff applied to the mid-phalanx of a finger with high optical sensitivity components and digital control systems. First, finger arterial blood pressure is measured by photoplethysmography placed inside the cuff. After an initial assessment of the pressure-diameter relation of the artery is made, arterial blood pressure is kept constant by a feedback system, which modulates finger cuff pressure across the measurement period. In this way, the system records the pressure that the finger artery would experience without the cuff. To correct the influence of hand height on the finger blood pressure measurement, the system measures the hydrostatic pressure difference between the hand and the heart (Martina et al. 2012).

From the finger recording, which is continuously calibrated by the Nexfin™ ‘Physiocal’ software, finger-to-brachial pressure reconstruction is performed using the software. Systolic (SBP) and diastolic (DBP) blood pressures were collected, while mean blood pressure (MBP) was determined as the time-weighted average of the recorded arterial blood pressure for each cardiac cycle. Its waveform was used to determine Modelflow® estimates of cardiac stroke volume (SV). The interval between R-waves on the ECG was used to calculate the heart rate (HR). Cardiac output (CO) was calculated as the product of HR and SV. Finally, peripheral vascular resistance (PVR) and the change in cardiac pressure with respect to time (dP/dt) were also gathered. For more information regarding the calculation and measurement methods by Nexfin™, the reader is referred to Martina et al. (2012).

### Cardiovascular variables windows of analysis

Given that continuous measurement of cardiovascular parameters was conducted with finger photoplethysmography, several time points were selected and gathered for further statistical analysis. Specifically, a 5-min pre-exercise time window was selected, with, at least, 2 min since the subject lied on the stretcher. From this, five means of one minute were calculated for all variables. During the 30-min recovery period, thirty means of one minute were computed for all the variables. Finally, a 5-min post-recovery time window was selected, starting 2 min after the end of the recovery, analysing five means of one minute for the variables.

For the posterior statistical analysis, five-minute pre-exercise values were averaged, from which the relative change, in percentage, of recovery and post-recovery variables for each subject and condition were calculated. The pre-exercise, baseline average represented 100%. Values below 100% denote a lower value than the pre-exercise mean, while values above 100% indicate a higher value. The relative change in each one-minute pre-exercise average to the five-minute pre-exercise average was also computed.

### Statistical analysis

Statistical analyses were performed using the Statistical Package for Social Science (V. 25.0, SPSS Inc., Chicago, IL). Descriptive statistics are shown as mean ± standard deviation unless otherwise stated. The normal distribution of the data for all measures was checked visually with histograms and by the Kolmogorov-Smirnoff test. Two-way repeated measures ANOVA was applied to determine differences in all variables collected between conditions (IPC vs. Sham) and time points of measurement, as well as possible conditions by time interactions. In case of detecting statistical differences (P < 0.05), Bonferroni *post-hoc* comparisons were also applied to search for possible specific differences. Effect size (Cohen *d*) was calculated to estimate the magnitude of the difference between group means, with d = 0.1, 0.3, 0.5, 0.7, and 0.9 reflecting small, medium, large, very large, and extremely large effect sizes, respectively.

## Results

Before the experimental recovery phase, IPC and Sham conditions showed similar results in the Watts (W) performed during the repeated sprint exercise, respectively (371.8 ± 22.2 vs. 372.4 ± 21.8 W; P = 0.986). The test re-test intraclass correlation between the exercises performed in both conditions (ICC 3, *1*) was 0.976, showing a similar exercise strain before starting the recovery phase.

### Blood pressure (SBP, DBP, and MBP)

**Fig. 1:**
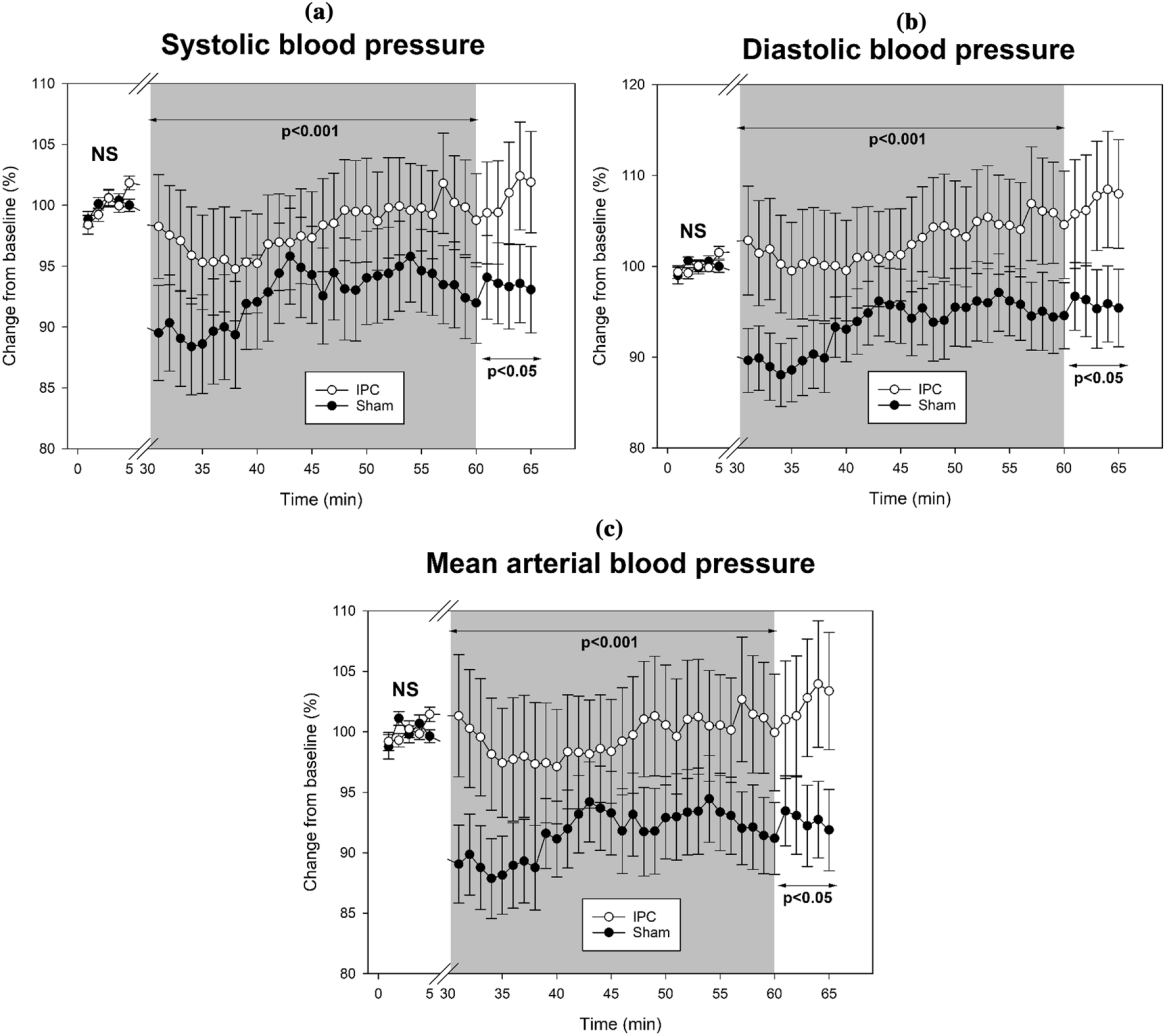
Changes in the *Blood pressure* parameters **a** systolic blood pressure, **b** diastolic blood pressure, and **c** mean arterial blood pressure before, during and after the sprint interval exercise in a sample of 16 trained subjects. Data shows the comparison between IPC () and Sham () conditions

Table 1 shows the changes in the *Blood pressure* values before the repeated sprint exercise, during the recovery phase, and after the recovery. In the baseline pre-exercise period, SBP, DBP, and MBP were not different between IPC and Sham conditions (P > 0.05) (Fig. 1).

**Table 1:** Changes in the *Blood pressure* values before the repeated sprint exercise, during the recovery, and after the recovery phase in a comparison between IPC and Sham conditions

			Pre-exercise	Recovery phase	Post-recovery
SBP (mmHg)	IPC	Absolute	121.5 ± 18.4	117.7 ± 19.2*	120.6 ± 16.5*
		Relative (%)	100 ± 2.8	98.0 ± 15.7	100.8 ± 16.6
	Sham	Absolute	129.3 ± 18.9	119.5 ± 26.5	120.9 ± 25.3
		Relative (%)	100 ± 2.3	92.6 ± 15.2	93.5 ± 13.3
DBP (mmHg)	IPC	Absolute	69.2 ± 8.6	70.1 ± 12.7*	73.0 ± 12.4*
		Relative (%)	100 ± 2.5	102.7 ± 21.8	107.2 ± 23.8
	Sham	Absolute	73.8 ± 12.1	68.5 ± 14.1	70.1 ± 13.0
		Relative (%)	100 ± 2.7	93.5 ± 15.4	95.9 ± 16.1
MBP (mmHg)	IPC	Absolute	87.6 ± 11.7	86.2 ± 14.9*	88.6 ± 14.0*
		Relative (%)	100 ± 2.5	99.5 ± 18.5	102.5 ± 19.3
	Sham	Absolute	93.7 ± 14.1	85.9 ± 19.5	86.8 ± 18.3
		Relative (%)	100 ± 2.7	91.6 ± 13.2	92.7 ± 12.5

Data shows absolute parameters and relative changes to “pre-exercise” in a sample of 16 trained participants. Results are expressed as mean ± standard deviation absolute values and relative change, in percentage, from the pre-exercise. Pre-exercise and post-recovery data represent 5-min periods, while recovery data represent the 30-min average values

*Significantly different from the Sham condition (p < 0.05)

During the experimental recovery phase, there were significant differences in SBP, DBP, and MBP between the IPC and Sham conditions comparing the relative changes to the pre-exercise values. The results show higher parameters during the 30-min recovery interval for the IPC condition compared to Sham, in SBP (98.0 ± 15.7% vs. 92.6 ± 15.2%; P < 0.001; *d* = 0.349), DBP (102.7 ± 21.8% vs. 93.5 ± 15.4%; P < 0.001; *d* = 0.487), and MBP (99.5 ± 18.5% vs. 91.6 ± 13.2%; P < 0.001; *d* = 0.492). In the post-recovery period, SBP, DBP, and MBP relative changes from baseline were closer to the homeostatic parameters in the IPC condition, in SBP (100.8 ± 16.6% vs. 93.5 ± 13.3%; P = 0.002; *d* = 0.485), DBP (107.2 ± 23.8% vs. 95.9 ± 16.1%; P < 0.001; *d* = 0. 556), and MBP (102.5 ± 19.3% vs. 92.7 ± 12.5; P < 0.001; *d* = 0.572).

In the comparison between sexes, during the experimental recovery phase, females exhibited closer values to pre-exercise than males in SBP (98.0 ± 0.8% vs. 93.1 ± 0.7%; P < 0.001), DBP (101.7 ± 1.0% vs. 95.3 ± 0.9%; P < 0.001), and MBP (98.6 ± 0.9% vs. 93.2 ± 0.8%; P < 0.001). The interaction between conditions (IPC and Sham) and sex (females and males) did not show significant differences in the evolution of SBP (P = 1.000), DBP (P = 1.000), and MBP (P = 1.000) during the recovery phase.

### Heart function (HR, SV, CO, and dP/dt)

**Fig. 2:**
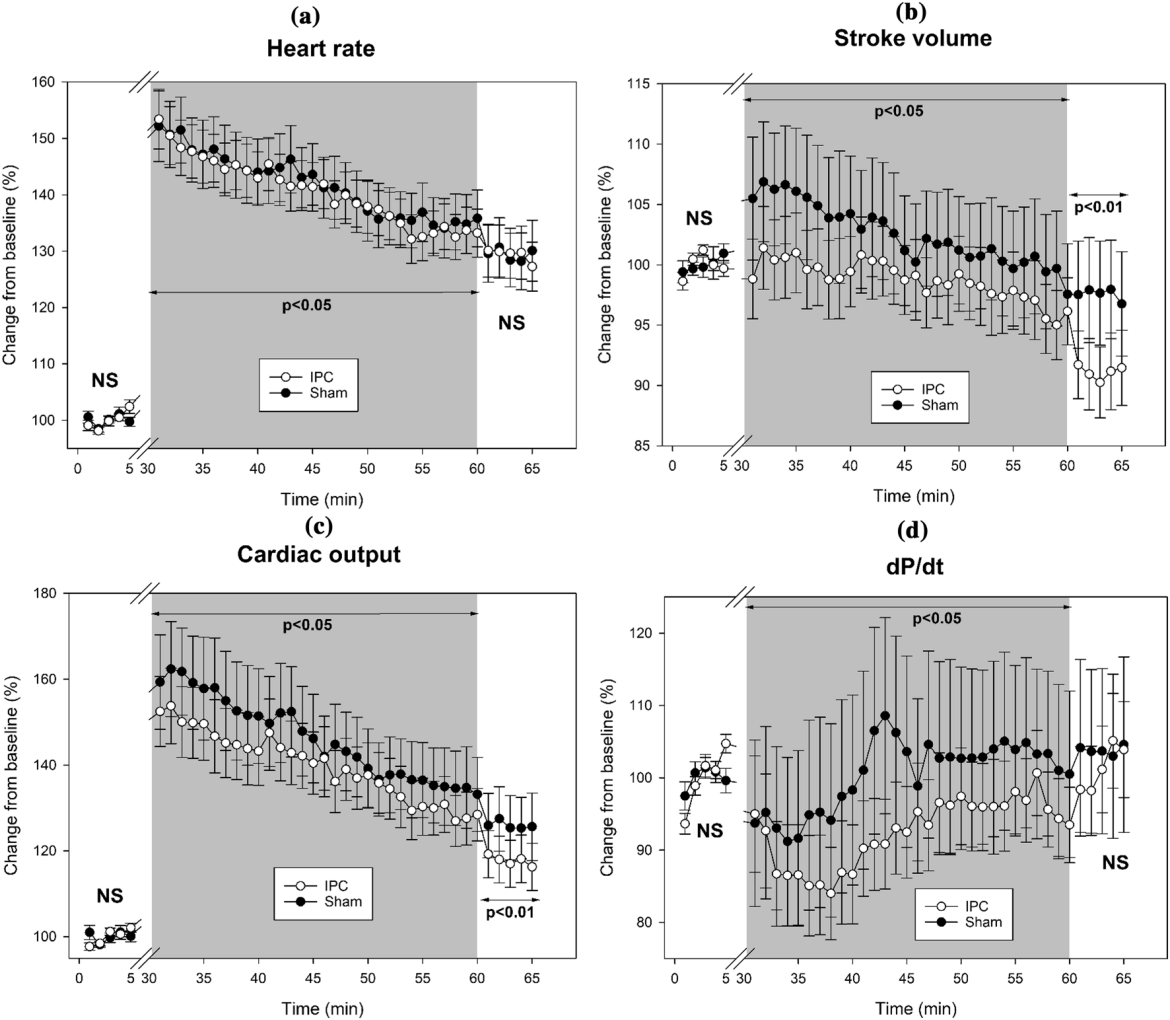
Changes in the *Heart function* parameters **a** heart rate, **b** stroke volume, **c** cardiac output, and **d** dP/dt before the repeated sprint exercise, during the recovery, and after the recovery phase in a sample of 16 trained subjects. Data shows the comparison between IPC () and Sham () conditions

Table 2 shows the changes in the *Heart function* parameters before the repeated sprint exercise, during the recovery phase, and after the recovery. In the baseline pre-exercise period, HR, SV, CO, and dP/dt were not different between IPC and Sham conditions (P > 0.05) (Fig. 2).

**Table 2:** Changes in the *Heart function* parameters before the repeated sprint exercise, during the recovery, and after the recovery phase in a comparison between IPC and Sham conditions

			Pre-exercise	Recovery phase	Post-recovery
HR (bpm)	IPC	Absolute	60.2 ± 11.0	83.7 ± 12.7*	77.1 ± 13.1
		Relative (%)	100.0 ± 3.7	140.6 ± 18.3	129.3 ± 17.9
	Sham	Absolute	62.5 ± 15.3	86.6 ± 15.1	79.1 ± 14.5
		Relative (%)	100.0 ± 3.6	141.7 ± 22.1	129.4 ± 19.9
SV (mL)	IPC	Absolute	105.4 ± 18.6	103.7 ± 21.0*	95.3 ± 18.5*
		Relative (%)	100.0 ± 2.4	98.7 ± 12.2	91.1 ± 11.7
	Sham	Absolute	104.2 ± 21.8	105.2 ± 22.2	99.9 ± 19.3
		Relative (%)	100.0 ± 3.6	102.5 ± 18.5	97.6 ± 16.8
CO (L/min)	IPC	Absolute	6.3 ± 1.5	8.7 ± 2.1*	7.3 ± 1.8*
		Relative (%)	100.0 ± 3.5	139.8 ± 30.0	117.7 ± 21.4
	Sham	Absolute	6.4 ± 1.9	9.1 ± 2.3	7.8 ± 1.8
		Relative (%)	100.0 ± 5.2	146.2 ± 40.2	125.9 ± 29.3
dP/dt (mmHg seg^−1^)	IPC	Absolute	907.1 ± 258.3	825.8.4 ± 311.4*	895.5 ± 273.6
		Relative (%)	100.0 ± 6.5	92.5 ± 25.8	101.4 ± 24.6
	Sham	Absolute	1089.8 ± 429.7	959.3 ± 294.6	1001.5 ± 298.0
		Relative (%)	100.0 ± 6.4	100.5 ± 48.9	103.8 ± 45.6

Data shows absolute parameters and relative changes to “pre-exercise” in the sample of 16 trained participants. Results are expressed as mean ± standard deviation absolute values and relative change, in percentage, from the pre-exercise. Pre-exercise and post-recovery data represent 5-min periods, while recovery data represent 30-min average values

*Significantly different from the Sham condition (p < 0.05)

During the experimental recovery phase, there were significant differences between the IPC and Sham conditions in HR, SV, CO, and dP/dt comparing the relative changes to the pre-exercise values. The results show a faster recovery during the 30-min recovery interval for the IPC condition compared to Sham, in HR (140.6 ± 18.3% vs. 141.7 ± 22.1%; P = 0.045; *d* = 0.054), SV (98.7 ± 12.2% vs. 102.5 ± 18.5%; P < 0.001; *d* = 0.243), CO (139.8 ± 30.0% vs. 146.2 ± 40.2%; P < 0.001; *d* = 0,180), and a slower recovery in dP/dt (92.5 ± 25.8% vs. 100.5 ± 48.9%; P < 0.001; *d* = 0.205) compared to Sham condition. In the post-recovery period, HR and dP/dt relative changes from baseline were not different between IPC and Sham conditions (P > 0.05). However, there were a greater decrease in the IPC condition for SV (91.1 ± 11.7% vs. 97.6 ± 16.8%; P = 0.003; *d* = 0.449), and CO (117.7 ± 21.4% vs. 125.9 ± 29.3%; P = 0.006; *d* = 0.320).

In the comparison between sexes, during the experimental recovery phase, females exhibited lower relative values compared to pre-exercise values than males in HR (133.2 ± 1.3% vs. 147.4 ± 1.1%; P < 0.001), SV (98.2 ± 0.9% vs. 102.5 ± 0.8%; P < 0.001), and CO (130.5 ± 2.1% vs. 152.7 ± 1.9%; P < 0.001). In dP/dt, no differences were shown between females and males (96.3 ± 2.1% vs. 96.6 ± 1.9%; P = 0.932). The interaction between conditions (IPC and Sham) and sex (females and males) did not show significant differences in the evolution of HR (P = 1.000), SV (P = 1.000), CO (P = 1.000), and dP/dt (P = 1.000) during the recovery phase.

### Peripheral vascular resistance (PVR)

**Fig. 3:**
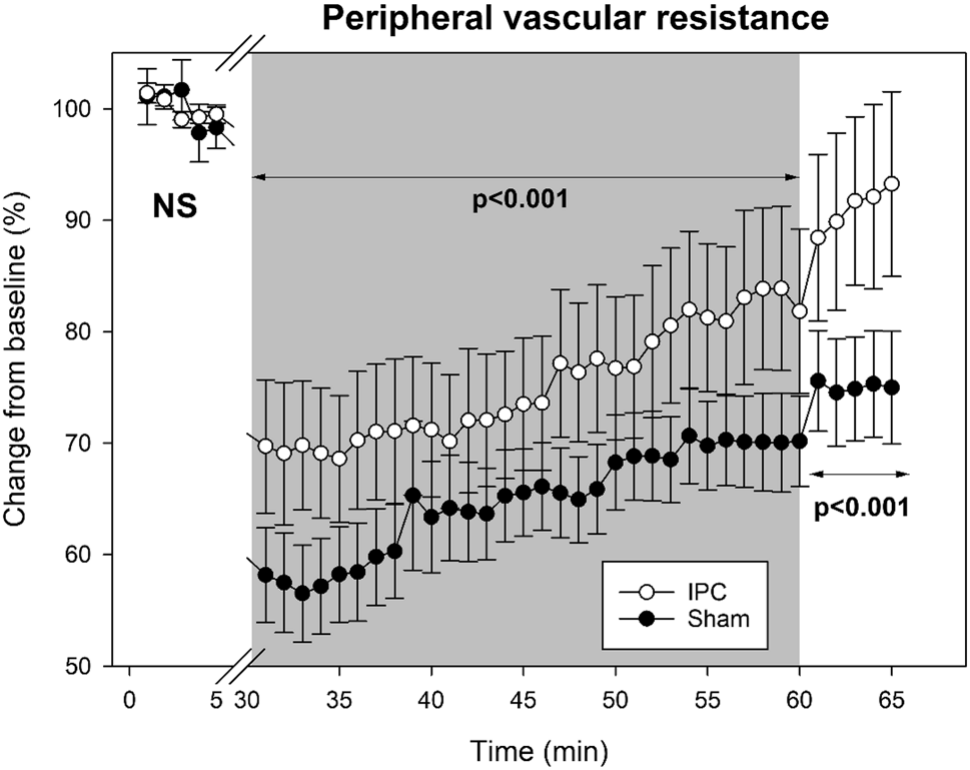
Changes in the *Peripheral vascular resistance* before the repeated sprint exercise, during the recovery, and after the recovery phase in a sample of 16 trained subjects. Data shows the comparison between IPC () and Sham () conditions

Table 3 shows the changes in the *Peripheral vascular resistances* values before the repeated sprint exercise, during the recovery phase, and after the recovery. In the baseline pre-exercise period, PVR was not different between the IPC and Sham conditions (p > 0.05) (Fig. 3).

**Table 3:** Changes in the *Peripheral vascular resistance* values before the repeated sprint exercise, during the recovery, and after the recovery phase in a comparison between IPC and Sham conditions

			Pre-exercise	Recovery phase	Post-recovery
PVR (dynas s cm^−5^)	IPC	Absolute	1186.8 ± 379.9	867.2 ± 332.3*	1049.6 ± 393.3*
		Relative (%)	100 ± 2.9	75.2 ± 25.5	91.1 ± 30.9
	Sham	Absolute	1328.8 ± 548.7	852.8 ± 469.1	978.1 ± 508.7
		Relative (%)	100 ± 8.8	64.8 ± 17.4	75.1 ± 18.6

Data shows absolute parameters and relative changes to “pre-exercise” in the sample of 16 trained participants. Results are expressed as mean ± standard deviation absolute values and relative change, in percentage, from the pre-exercise. Pre-exercise and post-recovery data represent 5-min periods, while recovery data represent the 30-min average values

*Significantly different from the Sham condition (p < 0.05)

During the experimental recovery phase, the PVR showed a closer recovery to pre-exercise baseline values in the IPC condition compared to Sham (75.2 ± 25.5% vs. 64.8 ± 17.4%; P < 0.001; *d* = 0.476). In the post-recovery period, the PVR response was also closer to the homeostatic baseline in the IPC condition (91.1 ± 30.9% vs. 75.1 ± 18.6%; P < 0.001; *d* = 0.627).

In the comparison between sexes, during the experimental recovery phase, females exhibited a higher relative value compared to pre-exercise than males in PVR (76.0 ± 1.2% vs. 65.4 ± 1.0%; P < 0.001). The interaction between conditions (IPC and Sham) and sex (females and males) did not show significant differences in the evolution of PVR (P = 1.000) during the recovery phase.

## Discussion

Intending to improve post-exercise recovery, intermittent pneumatic compression has lately gained popularity among sports practitioners. Although past research has gained insights into the local and systemic cardiovascular response, this is the first study, to our knowledge, to analyse the acute effects of IPC on cardiovascular physiology during the recovery from a strenuous maximal exercise.

The present study hypothesised that using IPC would accelerate the recovery of heart and circulatory parameters during the recovery phase. The main findings shows a faster recovery of HR, SV, CO, PVR, SBP, DBP, and MBP to pre-exercise values, and the slower recovery of dP/dt during the 30-min IPC-based recovery protocol compared to the Sham condition after a repeated sprint interval exercise.

### IPC—mechanism of action

The application of an external and intermittent pressure gradient in the limbs aims to increase the venous and arterial blood flow, as well as to ease the movement of interstitial fluid from the tissue to the lymphatic and blood vessels (Chen et al. 2001; Waller et al. 2006; Morris 2008). In this regard, intermittent pneumatic compression has been shown to increase velocity and volume flow in several deep and superficial leg veins, even more than leg elevation (Lurie et al. 2003).

This change in vessel haemodynamics depends on the phase of the moment of the cycle. IPC mechanical effects consist of deep vein compression, emptying the venous compartment, and increasing antegrade blood flow (Chen et al. 2001). Morris and Woodcock (2002) measured healthy subjects and others with peripheral arterial disease in supine and resting condition, observing that 10 s of thigh and calf compression at 60 mmHg reduced common femoral artery blood flow and increased retrograde (proximal) flow, while the subsequent 50 s of decompression increased the vessel outflow. In another study, Sheldon et al. (2012) observed a decrease in blood flow and arterial shear rate during compression, with the contrary effect during cuff deflation after an application of 120 mmHg intermittent pneumatic compression in the foot and calf of human subjects in an upright posture, In rats, Roseguini et al. (2010) showed how short cycles of lower leg intermittent compression (i.e., 2 or 4 s of compression and 2 to 16 s of decompression, at both 120 mmHg and 200 mmHg) induced femoral artery blood flow reduction or increase during compression and decompression, respectively.

In this way, it is thought that IPC could move blood from distal to central blood vessels, increasing venous return and enhancing cardiovascular function (Khan et al. 2021). According to Guyton’s model, venous return magnitude depends on the pressure difference between mean systemic filling pressure (P_msf_) -the forward flow coming from the venous system- and right atrium pressure, relative to resistance to venous return (Tansey et al. 2019; Persichini et al. 2022). Given that P_msf_ depends on blood volume status and vasomotor tone, changes in venous capacitance and vein blood volume could alter it (Persichini et al. 2022). This is the aim of several clinical techniques prompting a fluid challenge to the body, such as fluid administration in critically ill patients or passive leg raising, which increase P_msf_ and, depending on the preload responsiveness of the patient’s heart, can increase venous return (Guérin et al. 2015; Persichini et al. 2022).

### Blood pressure (SBP, DBP, and MBP)

IPC recovery, compared to the Sham condition, resulted in a faster return of SBP, DBP, and MBP to pre-exercise values, which was also observed during the post-recovery period. After a 1-h IPC application in a leg-dependent position, Sheldon et al. (2012) reported an increase in MBP. However, the immediate application of IPC in the upright position after light exercise did not alter the normal response of mean arterial pressure (MAP) (Zuj et al. 2018, 2019). Fanelli et al. (2008) observed an increase in MAP after a 30-min 40 mmHg IPC application in a resting, supine condition in healthy subjects.

Discrepancies between studies could include body positioning when applying pneumatic compression, resting or post-exercise recovery states, the type of device, its compression cycle and applied pressure. It could be argued that recovery in an upright or leg-dependent position could be different from supine, as the former implies a blood pooling in the dependent limbs (Romero et al. 2017), although Lurie et al. (2003) did not report wide differences in the mean peak velocity and volume flow increase in several superficial and deep leg veins when comparing a horizontal and 15° head-up position.

Blood pressure depends on cardiac inflow (cardiac output) and the ease of outflow (represented by systemic vascular conductance, the inverse of total peripheral resistance) (Romero et al. 2017). In this study, cardiac output was reduced when applying IPC compared to Sham, whereas PVR was increased. Following the assumption on the PVR determinants, the higher reduction in CO would have been compensated by the higher PVR, maintaining blood pressure values to guarantee tissue and organ perfusion (Iellamo 2001; Romero et al. 2017).

The physiological mechanism behind IPC application could be a modulation of sympathetic and/or parasympathetic activity (see *Heart function*), a compensation produced by the arterial baroreflex, increasing PVR to maintain blood pressure, and faster removal of local vasodilatory agents such as histamine (Romero et al. 2017). The mechanical-induced movement of blood flow would also provoke a higher strain and shear stress to endothelial cells, triggering biochemical responses such as secretion of tissue plasminogen activator, nitric oxide, prostacyclin, and others (Chen et al. 2001). It could also modify the molecular expression of several factors in compressed tissues (Sheldon et al. 2012). Future studies should observe the effects of IPC on blood pressure under different conditions and populations such as resting, warming up before exercise, or the effects of long-lasting interventions with patients with hypertension.

### Heart function (HR, SV, CO, and dP/dt)

The use of IPC-based recovery accelerates the HR, SV, and CO return to pre-exercise values, showing a significant reduction in post-exercise cardiovascular strain. In agreement with the present results, (Khan et al. 2021) showed that HR decline was higher when applying an 80-mmHg IPC in a supine position after a submaximal running exercise compared to the Sham condition. Other authors (Zuj et al. 2018, 2019) reported a HR decrease, SV increase, and CO increase or maintenance after 70-mmHg leg pneumatic compression application in a standing position during the diastolic cardiac cycle phase after a slight exercise. Contrarily, Martin et al. (2015b) did not observe a significant effect of 70-mmHg leg pneumatic compression on HR throughout the 30-min recovery after two Wingate tests.

Regarding dP/dt recovery dynamics, the lower values when applying IPC could mean that the heart had to produce a lower pressure for the time unit. In line with the other heart parameters mentioned earlier, this could result in a lower heart strain during IPC-based recovery.

Post-exercise recovery dynamics of cardiac variables depend on several factors, including recovery body position, prior exercise duration, and intensity, and active or passive recovery (Takahashi et al. 2005; Romero et al. 2017; Michael et al. 2017). Fanelli et al. (2008) showed a reduction in HR and body surface-normalized CO and maintenance of SV after a 30-min IPC at 40 mmHg in a resting supine state. Applying a 30 mmHg compression garment with continuous pressure in a resting state increases SV and CO, without modifying HR (Lee et al. 2020), while its application during a 60-min recovery from a fatiguing cycling exercise leads to SV and CO increases with a concurrent HR decrease in mid recovery (Lee et al. 2021). Contrarily, Bickel et al. (2011) reported no change in HR and an increase in CO and SV after a 15-min IPC at 50 mmHg. Reasons for these conflicting results could include different applied pressures, compression cycles, IPC-device design, or examination position, although measuring cardiovascular parameters in the left lateral decubitus position, as did Bickel et al. (2011), should be analogously compared to a supine position (Wieslander et al. 2019). Exercise imposes a cardiovascular demand to meet tissue-wide requirements, which is responded with an increase in sympathetic activity and a decrease in parasympathetic one (Kannankeril et al. 2004; Michael et al. 2017). Contrarily, cardiovascular recovery from exercise is represented by a starting fast phase, characterized by a high parasympathetic activity reactivation and the initiation of sympathetic activity withdrawal, followed by a slow, long-lasting phase of progressive increase of vagal activity and reduction of sympathetic tone (Arai et al. 1989; Kannankeril et al. 2004; Romero et al. 2017; Michael et al. 2017). In this study, the autonomous nervous system modulation would be related to the faster return to baseline values when using IPC, maintaining HR and CO above resting levels, while SV fell to pre-exercise levels with compression application. This could represent a higher vagal activity, a faster reduction in the sympathetic tone, or both (Kannankeril et al. 2004; Buchheit et al. 2009; Michael et al. 2017).

In fact, by using heart rate variability (HRV) and heart rate recovery (HRR) to assess autonomous nervous system activity, IPC application post-exercise and plasma volume gain have been associated with an increase in parasympathetic activity (Buchheit et al. 2009; Khan et al. 2021). Despite reporting a higher HRR when applying IPC, Rahman et al. (2022) did not observe a clear effect on HRV parameters after a submaximal treadmill exercise. Concurrently, IPC-induced metabolite elimination would diminish the activity of metaboloreflex, reducing, too, sympathetic activity (Iellamo 2001), and mechanical application of pressure with other methods, such as foam roller, has been shown to increase parasympathetic tone (Lastova et al. 2018). Future studies could explore the connection between the faster recovery of the studied cardiovascular parameters using IPC after an all-out exercise with further analysis of heart rate variability.

### Peripheral vascular resistance (PVR)

Peripheral vascular resistance is the mathematical inverse of vascular conductance, which, in turn, depends on the grade of vasodilation or vasoconstriction of vascular beds (Romero et al. 2017). After exercise, the characteristic vascular resistance decline represents a vessel vasodilation, which is hypothesized to be mediated by combined arterial baroreflex resetting, which drives a reduced sympathetic tone, and local vasodilatory mechanisms, especially histamine (Romero et al. 2017).

In this study, the application of IPC resulted in a faster return to pre-exercise levels during the intervention and post-recovery period compared with the Sham condition. In this regard, while Bickel et al. (2011) reported a decrease in PVR after 15 min of 50 mmHg IPC application in a resting, supine position, Fanelli et al. (2008), in healthy subjects, observed an increase after applying IPC for 30 min at 40 mmHg in the same conditions. Concurrently, Zuj et al. (2018, 2019) reported a higher vascular conductance after an IPC-based 2-min standing recovery from light, a monoarticular lower leg exercise and walking. Contrary to this, Sheldon et al. (2012) observed a decrease in vascular conductance after a 1 h IPC application in a leg-dependent position. Lee et al. (2021) also showed gradual post-exercise recovery of PVR, without an effect of continuously delivered 70 mmHg compression by leg garments.

In the present study, the associated faster return of PVR when using IPC could be related to arterial baroreflex resetting and local vasodilatory mechanisms (Romero et al. 2017). However, given that cardiac parameters return faster to pre-exercise values, representing a possible increase in vagal tone and/or a reduction in sympathetic activity, this would result in a lower PVR (Romero et al. 2017) contrary to our findings. Thus, it is plausible that an IPC-driven increase in blood volume and metabolite elimination could reduce local vasodilatory mechanism action, resulting in lower peripheral vasodilation and higher recovery of PVR. Concurrently, this could be associated with the maintenance of the higher PVR during the post-recovery period when using IPC.

### Differences between females and males

Regarding potential sex differences in the recovery dynamics, no statistical interactions were shown in the studied variables. Nonetheless, females exhibited closer values to baseline in the recovery phase for blood pressure (SBP, DBP and MBP) and PVR compared to males, as well as a lower relative values in HR, SV, and CO. This could display sex-related differences in cardiovascular physiology in terms of heart function or size, as well as hormonal disparities between sexes (Luczak and Leinwand 2009; Chaudhari et al. 2018). In fact, Carter et al. (2001) reported that females exhibit a faster return to basal values for CO and SV after a 5-min cycling exercise at 60% of predicted HR maximum compared to males, although females exhibited a higher decline in MBP contrarily to the present. In this sense, Yoo et al. (2017) observed, in older adults, how flow-mediated dilation response to a high and moderate intensity treadmill exercise was attenuated in men, but not in women. Further studies should involve more homogeneous, in terms of age and physical fitness, and larger sample size in order to examine deeper whether females and males respond differently to this intervention and other recovery strategies.

### Limitations

The present study is not free of limitations. First, IPC was applied following manufacturer instructions, at 80 mmHg pressure and a specific compression cycle. In addition, the studied subjects were physically active, but not elite or high-performance athletes. For these reasons, the extrapolation of the present results to other population groups and/or other pressures and compression cycles must be done carefully. The current findings of the study suggest the IPC accelerates cardiovascular variables, however, its application in assisting performance is non-existent, which makes it difficult to ascertain if the current cardiovascular modulation can provide beneficial effects on short-term performance. Also, the autonomous nervous system activity was not directly assessed by means of HRV records. Further investigations are warranted to comprehend potential links between cardiovascular recovery and assessment of sympathetic and parasympathetic modulation when applying IPC during post-exercise recovery. Finally, this work evaluated the effects of IPC in a short time window, while the recovery process can last several hours or days. Future studies aiming to evaluate the medium- and long-term effects of IPC post-exercise recovery are needed to understand the implications between short-term cardiovascular modulation, health, and performance.

## Conclusions

Driven by recent innovative technological advances in the field, the use of intermittent pneumatic compression is increasing among the physically active population. However, the perceived benefits reported by users have not been broadly studied from a physiological point of view. Although past research has gained insights into the local and systemic responses, this is the first study, to our knowledge, describing the benefits of intermittent pneumatic compression in the recovery of blood pressure, heart, and vasomotor function after a repeated sprint exercise. These results may have significant practical applications in sports medicine since the reduction in post-exercise cardiovascular strain may improve athlete’s recovery, particularly for athletes who perform multiple training sessions per day. Future studies should explore whether these adjustments match other physiological markers such as heart rate variability or blood biomarkers.

## Data Availability

The datasets that support this study are available from the corresponding author upon reasonable request.

## Abbreviations

BMI: Body mass index
CO: Cardiac output
DBP: Diastolic blood pressure
dP/dt: Change in arterial pressure over time
HR: Heart rate
HRR: Heart rate response
HRV: Heart rate variability
IPC: Intermittent pneumatic compression
MBP: Mean blood pressure
PVR: Peripheral vascular resistance
P_msf_: Mean systemic filling pressure
RSE: Repeated sprint exercise
SBP: Systolic blood pressure
SV: Stroke volume
